# Successful remission of advanced basosquamous cell carcinoma with cemiplimab monotherapy

**DOI:** 10.1016/j.jdcr.2026.02.030

**Published:** 2026-02-19

**Authors:** Dayna Gager, Bryan Anderson, Joseph Drabick

**Affiliations:** a Pennsylvania State College of Medicine, Hershey, Pennsylvania; b Department of Dermatology, Pennsylvania State Hershey Medical Center, Hershey, Pennsylvania; c Department of Medicine, Pennsylvania State Cancer Institute, Hershey, Pennsylvania

**Keywords:** antiprogrammed death receptor, basal cell carcinoma, basosquamous cell carcinoma, cemiplimab, immune checkpoint inhibitor, nonmelanoma skin cancer, programmed death receptor-1 inhibitor, squamous cell carcinoma

## Introduction

Basosquamous cell carcinoma (BSC) is a rare and aggressive subtype of basal cell carcinoma (BCC) that exhibits clinical and histological features of both BCC and squamous cell carcinoma (SCC). Current treatment guidelines remain largely extrapolated from BCC management despite evidence suggesting that BSC follows a more aggressive clinical course. Cemiplimab, a programmed death receptor-1 inhibitor, is approved for advanced SCC and refractory BCC, yet data on its efficacy in BSC remain scarce. To our knowledge, few cases of BSC remission with cemiplimab monotherapy have been previously reported. Here, we present a case of advanced BSC successfully treated with cemiplimab, highlighting its potential role in managing this challenging malignancy.

## Case

**Fig 1:**
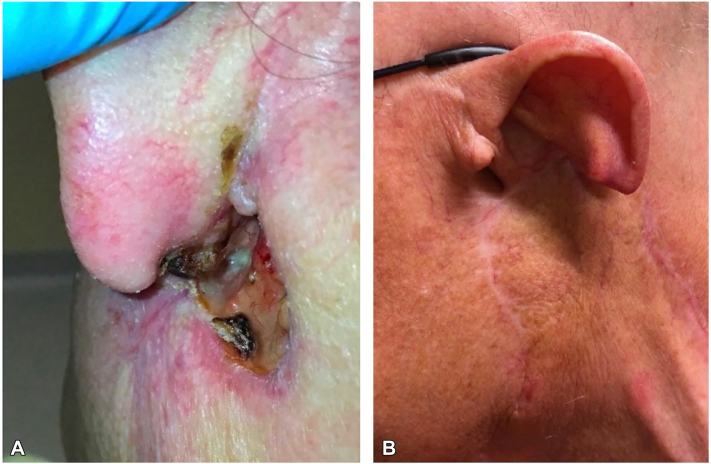
Left postauricular sulcus **(A)** before and **(B)** after cemiplimab treatment.

**Fig 2:**
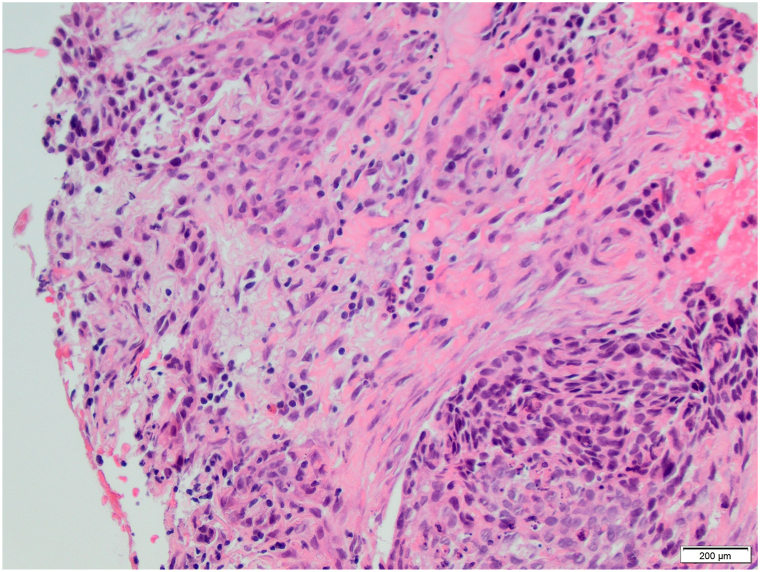
H&E stain, 20× magnification, showing the basosquamous cell carcinoma transition zone with areas of squamous differentiation on the top and left portion, in contrast to basophilic staining cells on the lower right. *H&E*, Hematoxylin and eosin.

**Fig 3:**
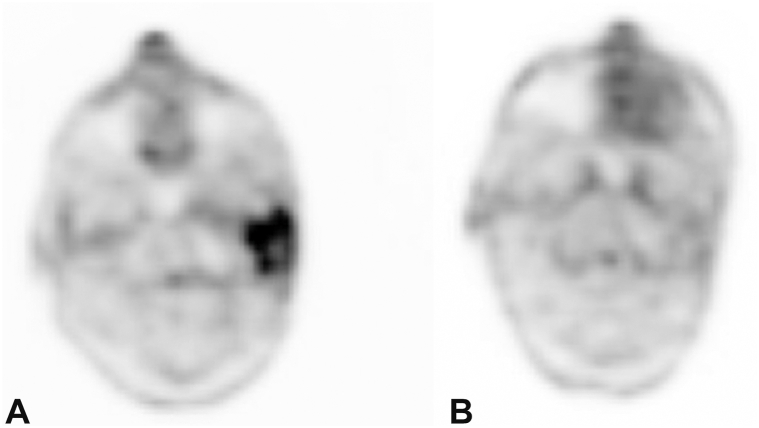
**A,** Baseline FDG-PET demonstrating a hypermetabolic mass involving the left parotid gland and **(B)** follow-up FDG-PET demonstrating sustained remission following cemiplimab therapy. *FDG-PET,* Fluorodeoxyglucose positron emission tomography.

A male in his 80s presented with a recurrent BCC of the left postauricular sulcus. The patient underwent multiple Mohs procedures, radiotherapy treatment and, despite initial improvement on vismodegib, the lesion ultimately recurred (Fig 1, *A*) with histologic conversion to BSC (Fig 2). A wide local excision with left partial auriculectomy and partial parotidectomy revealed infiltration of the left parotid gland with perineural invasion. The tumor was staged at pT3 and adjuvant radiotherapy was completed. At follow-up 6 months later, the patient had developed near complete left facial paralysis. A punch biopsy of the area showed metastatic BSC, diffusely positive for p63 and focally positive for Ber-EP4. Full-body fluorodeoxyglucose positron emission tomography (PET) revealed a hypermetabolic mass (3.5 × 3.1 × 6.1 cm) involving the superficial and deep lobes of the left parotid gland, extending inferiorly along the sternocleidomastoid muscle (Fig 3, *A*). Advancement of disease precluded further surgical intervention, and treatment was promptly initiated with cemiplimab infusions at 3-week intervals. Repeat PET imaging performed after 4 and 8 cycles demonstrated complete remission. Treatment was discontinued after 24 cycles following confirmation of sustained remission on follow-up PET imaging (Fig 3, *B*). One year later, he remains without disease recurrence (Fig 1, *B*).

## Discussion

BSC poses diagnostic and therapeutic challenges due to its ambiguous classification and histopathological overlap with both BCC and SCC. Clinically, it often presents as a nodular lesion resembling BCC before progressing to ulceration, while histologically it comprises BCC-like and SCC-like areas with a transition zone exhibiting features of both.^1^

Dermoscopy frequently reveals peripheral arborizing vessels and keratin masses.^2^ However, shave biopsies may be inadequate for diagnosis, as superficial sampling may only capture BCC features, missing deeper areas of squamous differentiation.^1^ As such, the true incidence of BSC may be underestimated, although retrospective reviews estimate it between 1.7% and 2.7% of non-melanoma skin cancers.^1^

Although the National Comprehensive Cancer Network classifies BSC as high-risk because of its estimated 5% to 10% metastatic potential, comparable to SCC, and its propensity for recurrence, there are currently no specific treatment guidelines for this entity.^3^ Standard treatment remains wide local excision or Mohs micrographic surgery; however, recurrence rates are significantly higher than that of both BCC and SCC underscoring the need for alternative treatment.^1^

Given the high frequency of sonic hedgehog pathway mutations in BSC, hedgehog inhibitors (HHIs), such as vismodegib and sonidegib, are often employed in cases of locally advanced or metastatic disease.^4^ However, resistance to HHIs is well documented, with secondary malignancies developing in some patients undergoing HHI therapy.^5^ Notably, the patient described in our case exhibited histologic conversion to a more aggressive phenotype following HHI therapy, suggesting a potential mechanism of resistance. Combination therapy with HHIs and itraconazole shows promise against resistance, but data are limited, and its efficacy in BSC remains unstudied.^6^

Immune checkpoint inhibitors (ICIs), such as cemiplimab, have emerged as an alternative approach for advanced NMSC. Cemiplimab, a programmed death receptor-1 inhibitor, has demonstrated efficacy in advanced SCC and, in 2021, received Food and Drug Administration approval as second-line therapy for advanced BCC following HHI failure or intolerance.^4^ Despite limited data regarding its role in BSC, the overlapping molecular and histologic features of BCC and SCC suggest a rationale for its use in this setting.

Only a few reports have documented successful treatment of BSC with ICIs. One case achieved remission with a combination of vismodegib and cemiplimab,^7^ another used cemiplimab in combination with adjunctive radiation therapy,^8^ and a third with cemiplimab monotherapy following sonidegib failure.^9^ Conversely, Proietti et al presented a case of BSC responding to sonidegib after cemiplimab failure, highlighting variability in treatment responses.^4^

Radiotherapy has been proposed as a potential adjunct to immune checkpoint blockade.^8^ While retrospective studies, such as that by Mager et al, have evaluated cemiplimab with radiotherapy in advanced BCC, no studies to date have specifically investigated the combination of cemiplimab and radiotherapy in BSC.^10^ Given the paucity of data, future research is warranted to explore the potential synergy between these modalities in BSC management.

## Conclusion

BSC represents a diagnostically and therapeutically challenging entity. Its high recurrence and metastatic potential necessitate treatment beyond surgery. Our case supports cemiplimab’s efficacy, although the role of ICIs in BSC remains incompletely characterized. Further studies are needed to delineate optimal treatment sequencing, combination strategies, and long-term outcomes as the landscape for advanced non-melanoma skin cancers evolves.

## Conflicts of interest

None disclosed.
